# Supplementary material for: Chronic Social Isolation Reshapes the Metabolome in Drosophila
Source: bioRxiv. 2026 Sep 30:2026.09.24.754212. Preprint. [Version 1] doi: 10.64898/2026.09.24.754212 (PMC13648389; doi:10.64898/2026.09.24.754212)
Supplement: Supplementary file 1 [file NIHPP2026.09.24.754212v1-supplement-1.pdf]

bioRxiv preprint doi: https://doi.org/10.64898/2026.09.24.754212; this version posted September 30, 2026. The copyright holder for this preprint (which was not certified by peer review) is the author/funder, who has granted bioRxiv a license to display the preprint in perpetuity. It is made available under a CC-BY 4.0 International license.

Supplementary Materials

Supplemetary Figure 1

A

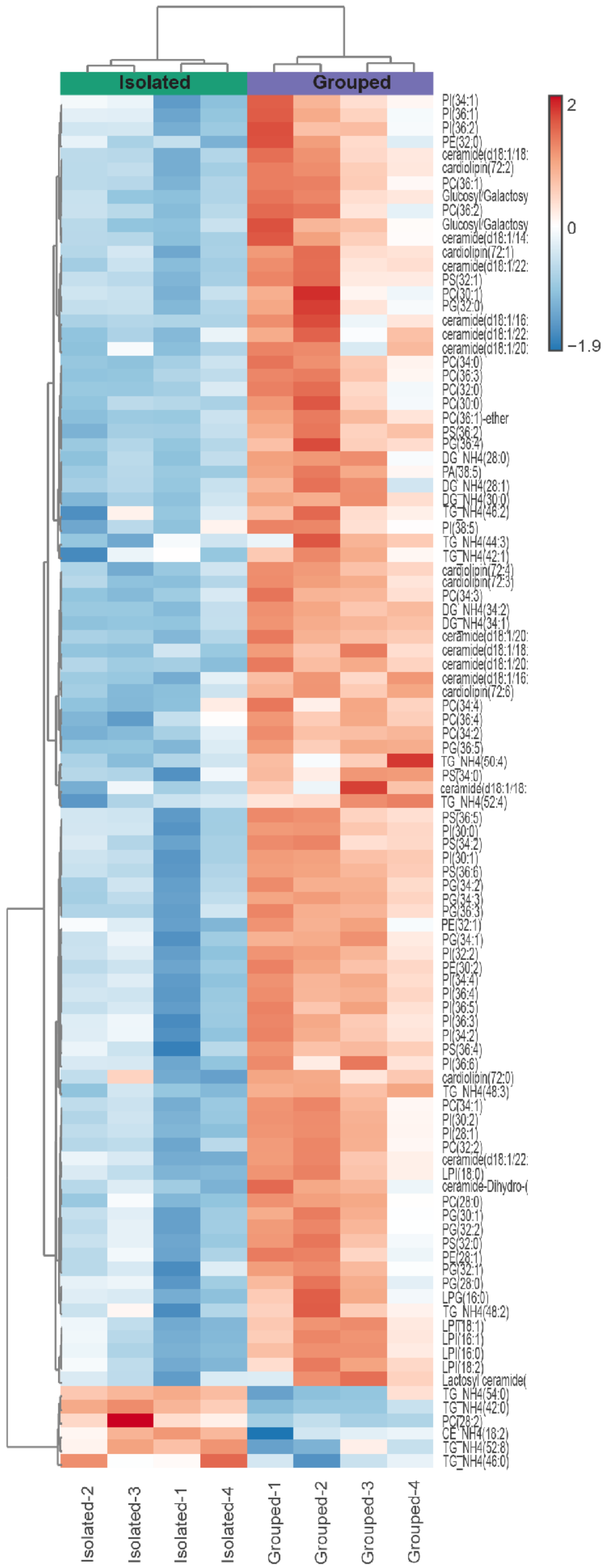

bioRxiv preprint doi: https://doi.org/10.64898/2026.09.24.754212; this version posted September 30, 2026. The copyright holder for this preprint (which was not certified by peer review) is the author/funder, who has granted bioRxiv a license to display the preprint in perpetuity. It is made available under a CC-BY 4.0 International license.

Supplementary Figure 1. Hierarchical clustering of the top 100 significantly altered lipids following chronic social isolation. Heatmap showing unsupervised hierarchical clustering of the 100 most significantly altered lipid species between group-housed and socially isolated 2202u flies after 7 days of housing. Columns represent individual biological replicates and rows represent lipid species. Lipid abundance is displayed as row-scaled Z-scores.

bioRxiv preprint doi: https://doi.org/10.64898/2026.09.24.754212; this version posted September 30, 2026. The copyright holder for this preprint (which was not certified by peer review) is the author/funder, who has granted bioRxiv a license to display the preprint in perpetuity. It is made available under a CC-BY 4.0 International license.

Supplemetary Figure 2

A

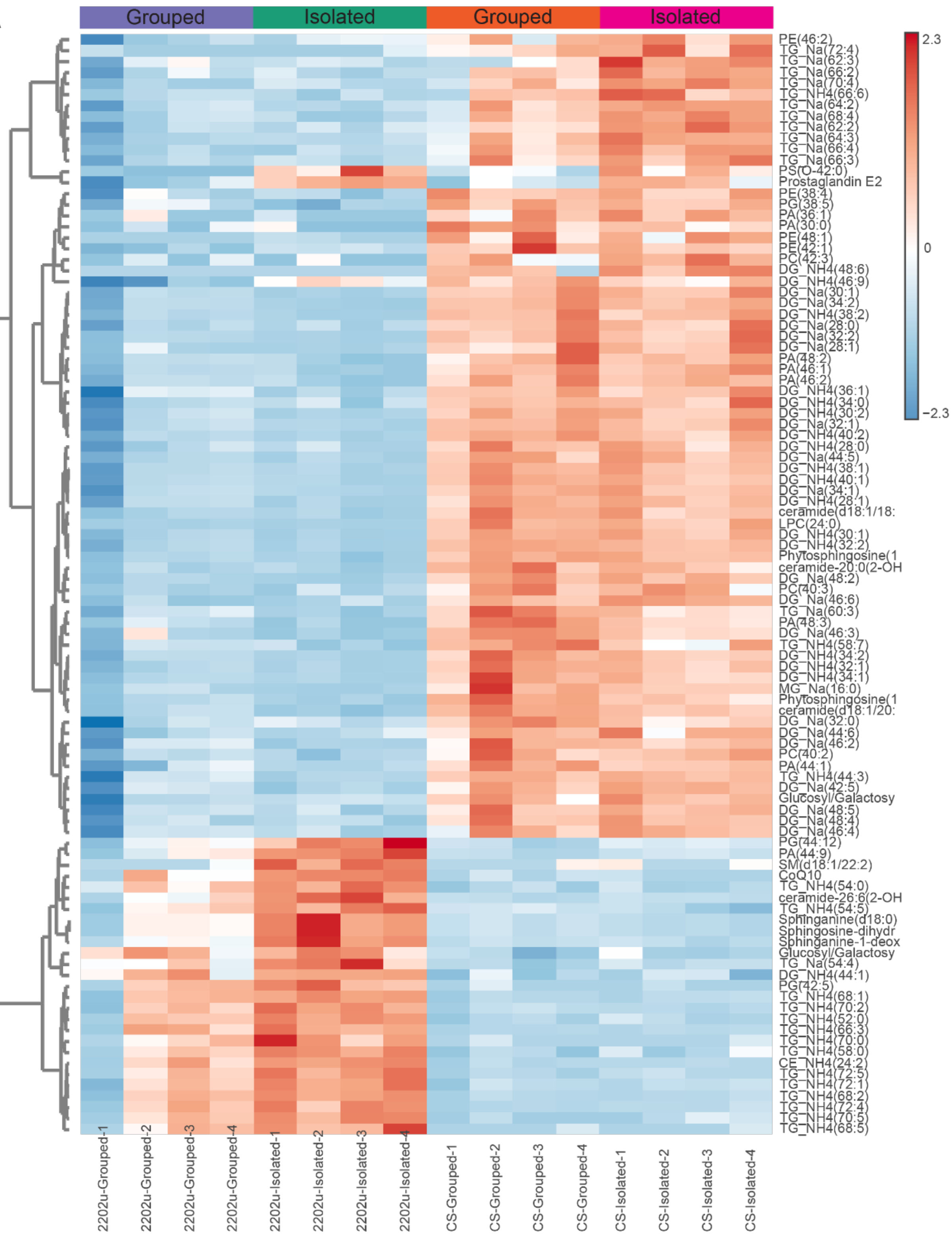

Supplementary Figure 2. Hierarchical clustering reveals distinct lipidomic profiles in 2202u and Canton-S flies. Heatmap showing unsupervised hierarchical clustering of

bioRxiv preprint doi: https://doi.org/10.64898/2026.09.24.754212; this version posted September 30, 2026. The copyright holder for this preprint (which was not certified by peer review) is the author/funder, who has granted bioRxiv a license to display the preprint in perpetuity. It is made available under a CC-BY 4.0 International license.

the top 100 differentially abundant lipid species from group-housed and socially isolated 2202u and Canton-S (CS) flies after 7 days of housing. Columns represent individual biological replicates and rows represent lipid species. Lipid abundance is displayed as row-scaled Z-scores.
